# Prognostic value of proadrenomedullin in severe sepsis and septic shock patients with community-acquired pneumonia

**DOI:** 10.1186/cc9696

**Published:** 2011-03-11

**Authors:** B Suberviola, A Castellanos, L García Astudillo, D Iglesias, F Ortiz Melon

**Affiliations:** 1 University Hospital Marques de Valdecilla, Santander, Spain

## Introduction

Community-acquired pneumonia (CAP) is the leading cause of death from infectious disease in western countries and supposes an important consumption of healthcare resources. Several studies suggest that proADM is possibly as good as validated severity scores in detecting critically ill patients with CAP and probably better than other biomarkers like procalcitonin (PCT).

## Methods

A single-centre prospective study between January 2009 and September 2009. Eligible patients were all consecutive adult patients, age 17 or older, admitted to the ICU with both a clinical and radiologic diagnosis of pneumonia as per Fine and colleagues, and meeting criteria for severe sepsis or septic shock. Venous blood samples were obtained at admission on the ICU and collected in tubes containing EDTA. After centrifugation, they were kept frozen at -80°C until assayed. MR-proADM, PCT and C-reactive protein (CRP) were measured in these samples.

## Results

In all cases, proADM values at ICU admission were pathological. ProADM consistently rose as PSI class advanced from II to V (*P *= 0.02). Differences across PSI class were not significant for CRP (*P *= 0.73) and PCT (*P *= 0.12). Median proADM levels were higher (*P *= 0.007) in hospital nonsurvivors (8.1 ± 9.2 nmol/l) versus survivors (3.0 ± 3.2 nmol/l). These differences were also significant with respect to ICU mortality (9.9 ± 10.4 vs. 3.2 ± 3.2 nmol/l; *P *= 0.001). The receiver-operating characteristic curve for proADM yielded an AUC of 0.72; better than the AUC for PCT and CRP (0.40 and 0.44, respectively) and similar to PSI (0.74). The optimal prognostic cut-off (maximum combined sensitivity and specificity) related to in-hospital mortality for proADM was 4.86 nmol/l, with a sensitivity of 0.53, specificity of 0.84, positive likelihood ratio of 3.39, negative likelihood ratio of 0.56, positive predictive value of 64.3 and negative predictive value of 77.1. Those patients with a proADM level higher than 4.86 nmol/l on ICU admission had an in-hospital mortality significantly higher than those with lower value.

## Conclusions

ProADM levels on ICU admission predict the severity and outcome of severe sepsis and septic shock CAP with a similar prognostic accuracy as the PSI and a higher prognostic accuracy compared with commonly measured laboratory parameters.

